# miRNA-195 expression in the tumor tissues of female Brazilian breast cancer patients with operable disease

**DOI:** 10.6061/clinics/2021/e2142

**Published:** 2021-01-11

**Authors:** Alexandre Cesar Vieira de Sales, Isaura Isabelle Fonseca Gomes da Silva, Matheus Carvalho Brito Leite, Leandro de Lima Coutinho, Renata Bezerra de Albuquerque Cavalcante Reis, Danyelly Bruneska Gondin Martins, José Luiz de Lima Filho, Fabrício Oliveira Souto

**Affiliations:** I Laboratorio de Imunopatologia Keizo Asami (LIKA), Universidade Federal de Pernambuco (UFPE), Recife, PE, BR; II Nucleo de Ciencias da Vida (NCV), Centro Academico do Agreste (CAA), Universidade Federal de Pernambuco (UFPE), Caruaru, PE, BR

**Keywords:** miRNA-195, Breast Cancer, Biomarker, Tumor Tissue, Neoadjuvant Chemotherapy

## Abstract

**OBJECTIVE:**

This study aimed to assess miRNA-195 expression in the tumor tissues from a cohort of Brazilian female breast cancer patients undergoing neoadjuvant chemotherapy (NAC) and evaluate its correlation with various clinicopathological markers.

**METHODS:**

Quantitative reverse transcription polymerase chain reaction (qRT-PCR) was used to evaluate the miRNA-195 expression in tumor tissues from a cohort of female breast cancer patients undergoing NAC. This expression was then correlated with the occurrence of several distinct breast cancer molecular subtypes and other clinicopathological variables.

**RESULTS:**

A total of 55 patients were included in this study, 28 (50.9%) of whom were treated using NAC. Tumor miRNA-195 expression was suppressed in breast cancer patients, regardless of their exposure to systemic treatments, histological grade, size, nodal status, and tumor-node-metastasis (TNM) staging. This was more pronounced in luminal and triple-negative patients, and patient’s response to NAC was correlated with an increase in miRNA-195 expression.

**CONCLUSION:**

miRNA-195 is downregulated in the tumor tissues of Brazilian breast cancer patients regardless of NAC exposure; this reinforces its role as a tumor suppressor and a potential biomarker for chemotherapy response.

## INTRODUCTION

Breast cancer is the most common cancer among women, and there are an estimated 2.1 million new cases of breast cancer annually. Breast cancer is also the leading cause of cancer-related deaths in over 100 countries (1), and despite significant evolution in our understanding of breast cancer carcinogenesis and its molecular pathways, the development of noninvasive diagnostic and prognostic biomarkers remains an unmet need. In fact, mammography is still considered the gold standard for the screening and early diagnosis of breast cancer in asymptomatic patients (2).

MicroRNAs (miRNAs) are small noncoding RNA molecules comprising between 19 and 23 nucleotides; they regulate gene expression at the posttranscriptional level (3). Accumulating evidence suggests that miRNAs play a crucial role in several physiological and pathological processes including metabolism, proliferation, differentiation, and apoptosis. Their dysregulation has been associated with a broad spectrum of diseases including Alzheimer’s dementia, Parkinson’s disease, diabetes, obesity, cardiovascular diseases, autoimmune disorders, and cancer (4,5).

miRNAs are differentially expressed in healthy and tumor cells and may even exhibit tissue-specific signatures among distinct types of cancer. These small regulators can either promote or suppress tumor development and progression by influencing various oncogenic processes including tumor initiation, promotion, malignant conversion, progression, and metastasis (6). In addition, miRNAs can be detected in the intracellular environment and in several body fluids, such as blood, urine, saliva, semen, and breast milk. This makes them a promising source of stable, noninvasive biomarkers for cancer diagnosis, prognosis, and treatment (7,8).

miRNAs have been extensively investigated as promising biomarkers for the diagnosis, prognosis, and treatment of breast cancer and other neoplasms (9). Currently, only three established biomarkers (ER, PR, and HER-2) are routinely evaluated for all newly diagnosed breast cancer patients (10). However, there is a high degree of heterogeneity in the identity of the miRNAs associated with different cancers and given that most appear to be fairly non-specific, their application as both diagnostic and prognostic markers remain limited (11).

miRNA-195 is one of these markers; it originates from intron 7 on chromosome 17p13.1 and has been described as a tumor-suppressor molecule that is frequently downregulated in a variety of cancers including hepatocellular, bladder, colorectal, ovarian, peritoneal, and breast cancer (12). miRNA-195 exerts its anti-cancer effect against breast cancer by targeting fatty-acid synthase (FASN), 3-hydroxyl 3-methyl glutaryl CoA reductase (HMGCR), acetyl-CoA carboxylase (ACACA), and insulin receptor substrate-1 (IRS1), inhibiting the proliferation, invasion, angiogenesis, and metastasis of breast cancer cells (13,14).

Among the most consistently described diagnostic biomarkers for breast cancer, miRNA-195 has been identified as one of the most specific regulatory markers in these tissues since it can be used to discriminate between breast cancer and other neoplasms as well as breast cancer patients from healthy controls (15).

Most studies have described the downregulation of miRNA-195 in the bloodstream, mainly in the serum and plasma, of breast cancer patients (16-18). However, there are some conflicting data describing miRNA-195 expression in breast cancer tumor tissues. Qattan et al. (19), Takur et al. (17), and Cecene et al. (20) reported the downregulation of miRNA-195 in breast cancer tissues, while Heneghan et al. (21) described its upregulation in these tissues.

This study was designed to evaluate the expression of miRNA-195 in the tumor tissues from a cohort of Brazilian female breast cancer patients undergoing neoadjuvant chemotherapy (NAC), and correlate this with a variety of clinicopathological features.

## MATERIAL AND METHODS

### Study cohort and clinical sample processing

This analytical cross-sectional study included 55 patients with operable breast cancer treated at the Hospital do Câncer de Pernambuco between May 2018 and July 2018. NAC was offered to 28 of these patients and the protocol selection was performed at the discretion of the assistant physician. The inclusion criteria for this study included histologically confirmed invasive breast cancer and tumor-node-metastasis (TNM) with clinical staging between I and III (localized disease); the exclusion criteria included past treatments for cancer, neurological disorders, or autoimmune diseases. The clinicopathological and relevant demographic data for each of the patients were documented prospectively in our main breast cancer database. Cancer staging was performed according to the 8^th^ edition of the American Joint Committee on Cancer TNM criteria (22) and breast cancer molecular subtypes were defined using immunohistochemistry-based analysis of estrogen, progesterone, and HER-2, and Ki-67 receptor staining (23).

Healthy breast tissue samples from nine women who had undergone plastic surgery for cosmetic purposes were collected and used as the control samples. All tissue samples were obtained from surgical specimens, and a fragment up to 0.5 cm in thickness was submerged in five volumes of RNAlater™ (Qiagen, MD, USA) at room temperature for 2 h (to allow the solution to thoroughly penetrate the tissue) and then stored at -80°C for total RNA extraction.

### Ethical considerations

Approval and written informed consent were obtained from all study participants for the use of their blood, healthy breast tissues, and breast cancer tissue samples for research purposes. The study protocol was reviewed and approved by the Ethics Research Committee of the Hospital do Câncer de Pernambuco (approval number: CAAE 75061717.3.0000.5205) and was carried out in accordance with the Declaration of Helsinki.

### Tissue RNA extraction and DNase treatment

RNA extraction was performed using TRIzol^®^ (Invitrogen, Life Technologies, UK) on both healthy breast (control group) and breast cancer tissues. Breast glandular and tumor tissues were carefully dissected from the surrounding fatty tissue prior to TRIzol^®^ treatment. TRIzol^®^ reagent (1 mL) was added to 50-100 mg of tissue; the tissues were then homogenized using a mechanical tissue homogenizer at 0°C. RNA was treated with DNase using a commercially available kit (Ambion, UK), according to the manufacturer's recommendations; after RNA extraction, the RNA concentration was determined using a NanoDrop spectrophotometer (NanoDrop ND1000; Thermo Fisher Scientific, USA). RNA integrity analysis was performed using gel electrophoresis and total RNA was stored at -80°C.

### cDNA synthesis and gene expression

Aliquots of 10 ng of total RNA were used to produce cDNA using the TaqMan MicroRNA Reverse Transcription Kit (Applied Biosystems, USA) and 5X RT human TaqMan MicroRNA Assay primers (hsa-miR-195-5p: Assay ID_ 000494; hsa-miR-16: Assay ID_ 000391) according to the manufacturer’s instructions. The reaction was performed in a Veriti thermal cycler (Applied Biosystems, USA) for 30 min at 16°C, followed by 30 min at 42°C and an additional 5 min at 85°C.

Quantitative reverse transcription PCR (qRT-PCR) was performed using a 7500 Real Time PCR system (Applied Biosystems, USA) and TaqMan™ Universal PCR Master Mix (Applied Biosystems, USA), 20X primers, and nuclease-free water in a total reaction volume of 10 μL. The cycling process was set up as follows: 50°C for 2 min and incubation at 95°C for 10 min, followed by 45 cycles of 95°C for 15 s and 60°C for 1 min. The threshold standard deviation (SD) for intra-assay and inter-assay replicates was 0.3.

All samples were analyzed in triplicate using the ABI 7500 platform (Applied Biosystems, USA), and quantification cycle (C_q_) values were calculated using SDS 1.4 software (Applied Biosystems).

### Data processing and statistical analysis

qRT-PCR data was normalized using the 2^-ΔΔCq^ method (24). C_q_ values from the target, miRNA-195, were subtracted from the C_q_ values for the reference gene, miRNA-16, and the subsequent ΔΔC_q_ value was calculated for malignant tumors using the average of the control ΔC_q_ values. The expression values are represented as the ΔΔC_q_ value on a log_2_ scale. Statistical analysis was performed using the nonparametric Mann-Whitney U test, with a significance level of *p*≤0.05. All analyses were performed using GraphPad Prism version 7.00 (GraphPad Software, La Jolla, CA, USA).

The normality of the data was evaluated using the Shapiro-Wilk test. Variance homogeneity among the groups was assessed using the Levene test and numerical variables are described as the means±standard error values, while the categorical variables are shown as relative frequencies.

Group comparisons were performed using the generalized linear model and we used the Gamma model because of the nonparametric distribution of the data. When significance was reached, we conducted a *post hoc* analysis for single comparisons and Bonferroni correction for multiple comparisons. To analyze the relationship between miRNA-195 and tumor size, we calculated the Spearman’s rank correlation coefficient.

Differences with *p* values≤0.05 were considered statistically significant in all cases.

## RESULTS

The clinical and pathological characteristics of the breast cancer patients are summarized in Table 1.

The average age of the control group was 37.8±2.4 years and when we evaluated the breast cancer molecular subtypes we noted that the HER-2 group included 10 patients with luminal hybrid tumors (positive for estrogen and/or progesterone and HER-2 receptors) and two patients with HER-2 overexpression.

### Differences in tissue miRNA-195 expression between breast cancer patients and healthy control samples

**Figure 1:**
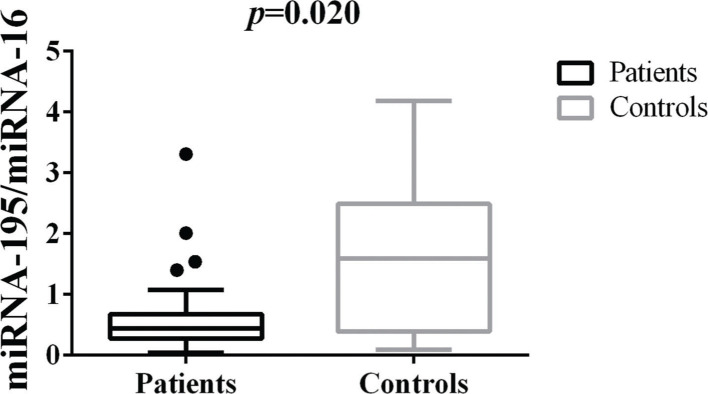
Patterns of miRNA-195 Expression in Breast Cancer Tissues. miRNA-195 expression in tumor (N=55) and healthy breast tissues (N=9). miRNA-195/miRNA-16 expression ratios were calculated using the 2^-ΔΔCt^ method. The expression values are presented as the ΔΔCq value on a log_2_ scale. Statistical analysis was performed using a generalized linear model and the data are presented as the mean±SE values. The black dots represent the outliers.

The comparative analysis of miRNA-195 expression in breast cancer and healthy breast tissues demonstrated a marked reduction in miRNA-195 expression in all the breast cancer patients included in this study, regardless of previous chemotherapy treatment (Figure 1).

### Tissue miRNA-195 expression according to breast cancer molecular subtype and other prognostic factors

When we analyzed miRNA-195 expression in tumor tissue samples stratified according to their breast cancer molecular subtype all, except the HER-2 samples, experienced some degree of downregulation. It is important to highlight that this decrease was more pronounced in the triple-negative breast cancer (TNBC) patient subgroup. This analysis suggests that there is a significant difference in miRNA-195 expression between the HER-2-positive and TNBC subgroups.

miRNA-195 was also shown to be downregulated irrespective of the histological grade when compared to the control group. This suppression was more pronounced in the ‘grade 3’ group and when intergroup analysis was performed for this clinicopathological variable, we observed a significant difference in miRNA-195 expression between ‘grade 3’ and ‘grade 2’ samples (Table 2).

When we evaluated the relationship between TNM breast cancer staging and miRNA-195 tissue expression, we were able to show that there was a significant decrease in miRNA-195 expression in clinical stages I, II, and III, when compared with that in subjects from the control group. Patients with metastatic disease (stage IV) were not included in this study.

**Figure 2:**
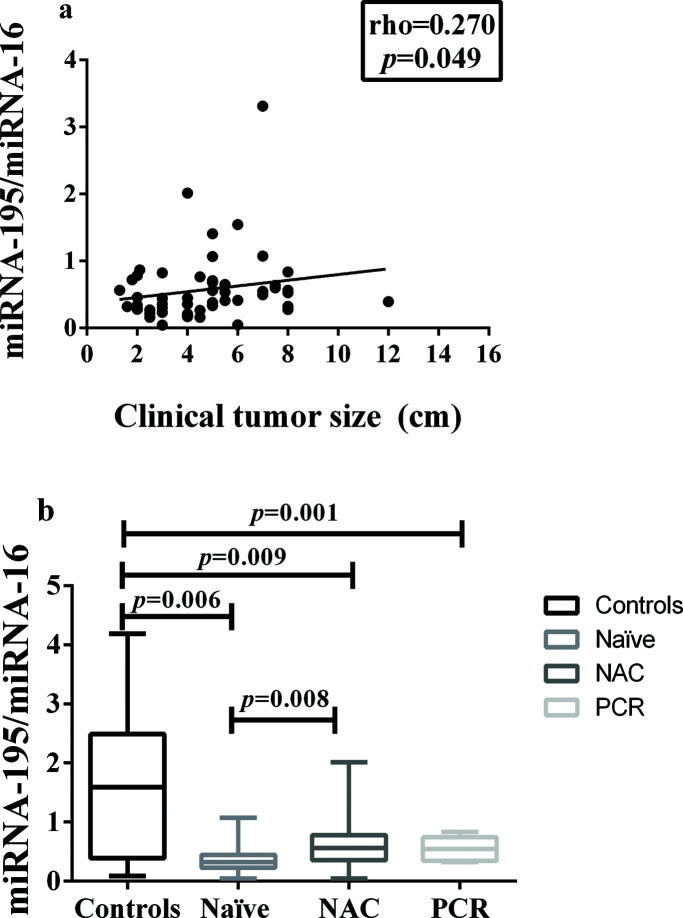
Correlation between miRNA-195 Expression and Tumor Size or Response to Neoadjuvant Chemotherapy. **a**) Correlation between miRNA-195 expression and tumor size (cm) (Spearman’s rank correlation coefficient). **b**) miRNA-195 expression following exposure to NAC (or not) and achievement of PCR (the miRNA-195/miRNA-16 ratios were calculated using the 2^-ΔΔCt^ method). The expression values are presented as the ΔΔCq values on a log_2_ scale (generalized linear model). The data are presented as the means±SE values. PCR, pathological complete response; NAC, neoadjuvant chemotherapy; Naïve, chemotherapy- naïve.

In addition, miRNA-195 was downregulated in all the breast cancer groups, regardless of the status of the axilla, and there was a weak positive correlation between tumor size and miRNA-195 expression (Figure 2a).

These results are all summarized in Table 2.

### miRNA-195 expression in breast cancer tumor tissues following NAC

The clinical and pathological characteristics of the 28 patients treated with NAC are summarized in Table 1.

The average tumor size for these patients before treatment was 5.75±0.43 cm, and the pathological size of the surgical specimens was 2.89±0.41 cm; a total of five of these patients achieved pathologic complete response in the breast and axilla: three HER-2-positive patients and two luminal B patients.

A comparison of the miRNA-195 expression levels among the patients in these three groups, namely: chemotherapy-naïve, neoadjuvant-treated, and pathologic complete response, revealed a significant reduction in miRNA-195 expression in all the cancer patients, compared to the controls. In addition, there was a significant difference in miRNA-195 tissue expression between the chemotherapy-naïve and neoadjuvant-treated groups, which presented with decreased and increased miRNA-195 expression, respectively (Figure 2b).

The analysis of miRNA-195 expression in tissue samples from molecular subtypes luminal A, luminal B, HER-2-positive, and TNBC focused on the chemotherapy-naïve and -treated patients and showed that there was a significant difference in miRNA-195 expression between subjects from these four subgroups. However, no intergroup differences were detected between the chemotherapy-naïve patients and those previously treated with chemotherapy.

When we evaluated miRNA-195 tissue expression in the various histological grades, we were able to demonstrate a significant reduction in miRNA-195 expression in subjects with all three grades of cancer, compared to the controls. However, there was no difference in its expression between the chemotherapy-naïve and -treated patient groups. This result was analogous to that observed for the TNM breast cancer stages.

There were also no significant differences in terms of lymph node involvement between the chemotherapy-naïve, neoadjuvant-treated, and control groups. These results are summarized in Table 3.

## DISCUSSION

The breast cancer patients included in this study mirror the clinical and epidemiological distribution of this disease in Brazil and the rest of the world (25). Conversely, contrary to the trend in developed countries to diagnose breast cancer in its initial stages, most patients included in this study received their diagnosis at more advanced stages, which unfortunately reflects the specific practices of the public health system in this country (26).

More than 90% of the cases were diagnosed with invasive carcinoma of a non-specific type, which is consistent with the findings described in the literature (27). However, when we evaluated the molecular subtype distribution of these breast cancer patients we observed that 28 (50,9%) of these cases were classified as luminal B cases, which is higher than the 15-20% reported in the literature (28).

This study demonstrated that miRNA-195 was downregulated in the tissues of breast cancer patients, when compared to the control, regardless of their exposure to NAC, thereby reinforcing its designation as a tumor-suppressor miRNA. Several studies have reported the suppression of miRNA-195 expression in breast cancer tissues and circulating fluids, especially the serum and plasma (16-20).

Heneghan et al. (15) described miRNA-195 as a specific biomarker for breast cancer diagnosis following their evaluation of 65 breast cancer tissues and 83 whole blood samples. In this study, the authors reported a concordant miRNA-195 overexpression in both tissue and whole blood samples. In another study, these same authors described miRNA-195 as a breast cancer-specific circulating biomarker as it consistently identifies early-stage breast cancer patients, distinguishing them from patients with other cancer types, healthy controls, and patients with benign breast lesions (21).

It is important to highlight that miRNA-195 expression significantly increased in the tumor tissues of NAC-treated patients who achieved clinical and pathological response to treatment, indicating a possible reconstitution of miRNA-195 expression as an effect of tumor destruction and surrounding tissue regeneration. This finding aligns with the findings described in the study by Fràres et al. (29). In their experiments, they demonstrated that the levels of miRNA-34a and miRNA-122 significantly increased in both the plasma tumor tissues of breast cancer patients following their clinical response to NAC.

miRNA-195 expression in the tissues of patients with different breast cancer molecular subtypes was suppressed in all groups, but was significant for the luminal A, luminal B, and TNBC subgroups, with a more prominent response in the TNBC subgroup. These findings were consistent with the results of studies by Chang et al. (30) and Qattan et al. (19), which consistently demonstrated reduced miRNA-195 expression in the tumor tissues of breast cancer patients. However, only eight patients (14,6%) were classified as having TNBC. Thus, this reduced miRNA-195 expression might be attributed to a reduced sample size, and this finding must be further explored.

Although the levels of miRNA-195 were suppressed in the tumor tissues of HER-2-positive breast cancer patients compared to those in the controls, it was not statistically significant. This result may be explained by the reduced sample size in this study and warrants further investigation in a larger cohort. The luminal A and B breast cancer subtypes exhibited reduced miRNA-195 expression, and no differences between the chemotherapy-treated and treatment-naïve patients was observed, which is consistent with the results of previous reports (19,20).

miRNA-195 was downregulated in all samples regardless of TNM stage, histological grade, molecular subtype, and nodal involvement, compared to the control and did not demonstrate any significant correlation with more advanced stages, poorly differentiated tumors, or axillary tumor burden. Thus, these findings do not reproduce the observations described in previous reports, suggesting an inverse correlation between miRNA-195 expression and these prognostic factors (31,32).

Nevertheless, it is important to mention the potential limitations of this study. First, the patients and subjects in the control groups were not age-matched; this may present a bias in our data, because the healthy controls were younger than the breast cancer patients. Finally, the ratio between malignant and nonmalignant cells in our tumor tissue samples was not evaluated and this may impact our interpretation of results, as we have no measure of tumor purity.

## CONCLUSION

Our study serves to further support the role of miRNA-195 as a tumor-suppressor miRNA; its expression was found to be downregulated in the tumor tissues of a Brazilian cohort of female breast cancer patients, regardless of previous exposure to systemic chemotherapy, breast cancer molecular subtype, and other prognostic factors, including TNM staging, tumor size, histological grade, and lymph node involvement. In addition, we demonstrated that miRNA-195 expression increased in patients who experienced a response to NAC treatment, indicating a possible reconstitution of miRNA-195 expression. Our future research will be focused on further exploring the simultaneous expression of miRNA-195 in both the tumor tissues and circulation.

## Figures and Tables

**Table 1:** The clinical and pathological characteristics of the study participants.

Variables	All patients (n=55)	Chemo naïve (n=27)	NAC (n=28)	*p*
Mean±standard error				
Age (y.o.)	54.2±1.9	54.3±2.8	48.1±2.1	0.001
Pathological tumor size (cm)	3.44±0.31	4.00±0.44	2.89±0.41	0.087
Clinical tumor size (cm)	4.67±0.31	3.56±0.37	5.75±0.43	0.001
Frequency n (%)				
Histologic types				0.589
Tubular carcinoma	1 (1.9)	1 (3.7)	-	
NST	52 (94.5)	25 (92.6)	27 (96.4)	
ILC	2 (3.8)	1 (3.7)	1 (3.6)	
Molecular subtypes				0.216
Luminal A	7 (12.7)	6 (22.2)	1 (3.6)	
Luminal B	28 (50.9)	12 (44.5)	16 (57.1)	
HER-2	12 (21.8)	5 (18.5)	7 (25.0)	
Triple-negative	8 (14.6)	4 (14.8)	4 (14.3)	
TNM staging				0.002
I	9 (16.4)	8 (29.6)	1 (3.6)	
II	29 (52.7)	16 (59.3)	13 (46.4)	
III	17 (30.9)	3 (11.1)	14 (50.0)	
Grade				0.338
1	4 (8.4)	3 (11.1)	1 (4.3)	
2	22 (45.8)	14 (51.9)	9 (39.1)	
3	22 (45.8)	10 (37.0)	13 (56.5)	
Lymph node involvement				0.995
None	35 (63.6)	17 (63.0)	18 (64.3)	
1 to 3	14 (25.5)	7 (25.9)	7 (25.0)	
≥4	6 (10.9)	3 (11.1)	3 (10.7)	

Invasive carcinoma of no special type (NST); Invasive lobular carcinoma (ILC); Overexpression (HER-2, HER-2) ; Tumor-node-metastasis breast cancer staging (TNM); Neoadjuvant chemotherapy (NAC).

**Table 2:** miRNA-195 expression stratified according to prognostic factors.

Breast cancer prognostic factors	miRNA-195/miRNA-16
Molecular subtype	
Control	1.64±0.41
Luminal A	0.56±0.38*
Luminal B	0.44±0.41*
HER-2	0.61±1.40‡
Triple-negative	0.31±0.28*
*p-*value	<0.001
TNM staging	
Control	1.64±0.41
Stage I	0.44±0.11*
Stage II	0.64±0.89*
Stage III	0.52±0.90*
*p-*value	<0.001
Grade	
Control	1.64±0.41
Grade 1	0.48±0.18*
Grade 2	0.71±0.11*
Grade 3	0.44±0.70* †
*p-*value	<0.001
Lymph node involvement	
Control	1.64±0.41
None	0.61±0.08*
1 to 3	0.50±0.10*
≥4	0.50±0.15*
*p-*value	<0.001

Values are presented as the means±standard error values.

* Significant difference compared to the control samples (*p*<0.05).

‡ Significant difference compared to triple-negative samples (*p*<0.05).

† Significant difference compared to grade 2 samples (*p*<0.05).

**Table 3:** Prognostic factors and miRNA-195 expression in patients treated with neoadjuvant chemotherapy.

Breast cancer prognostic factors	Control	Naïve	NAC	*p*
Molecular subtypes				<0.001
Control	1.64±0.41	-	-	
Luminal A	-	0.50±0.15*	-	
Luminal B	-	0.44±0.09*	0.56±0.11*	
HER-2	-	0.80±0.25	0.93±0.25	
Triple-negative	-	0.21±0.09*	0.42±0.13*	
TNM staging				0.001
Control	1.64±0.41	-	-	
Stage I	-	0.41±0.11*		
Stage II	-	0.52±0.10*	0.52±0.10*	
Stage III	-	0.60±0.25*	0.49±0.10*	
Grade				<0.001
Control	1.64±0.41	-	-	
Grade 1	-	0.48±0.21*		
Grade 2	-	0.61±0.12*	0.88±0.22*	
Grade 3	-	0.33±0.08*	0.53±0.11*	
Lymph node involvement				0.614
Control	1.64±0.41			
None	-	0.50±0.18	0.59±0.11	
1 to 3	-	0.46±0.08	-	
≥4	-	0.56±0.26	0.44±0.10	

Values are presented as the means±standard error values.

* Significant difference compared to the control (*p*<0.05). Tumor-node-metastasis breast cancer staging (TNM)
